# Supplementary material for: Pyruvate Dehydrogenase Kinase 1 inhibition mediated oxidative phosphorylation enhancement in cartilage promotes osteoarthritis progression
Source: BMC Musculoskelet Disord. 2023 Jul 20;24:597. doi: 10.1186/s12891-023-06585-6 (PMC10357736; doi:10.1186/s12891-023-06585-6)
Supplement: Supplementary file 1 — Additional file 1. [file 12891_2023_6585_MOESM1_ESM.zip › BP.Gsea.1653623667859/GOBP_REGULATION_OF_ENDOTHELIAL_CELL_DIFFERENTIATION.html]

Details for gene set GOBP\_REGULATION\_OF\_ENDOTHELIAL\_CELL\_DIFFERENTIATION[GSEA]

|  || Dataset | 55457\_collapsed\_to\_symbols.cls.cls #OA\_versus\_normal.cls.cls #OA\_versus\_normal\_repos |
| Phenotype | cls.cls#OA\_versus\_normal\_repos |
| Upregulated in class | normal |
| GeneSet | GOBP\_REGULATION\_OF\_ENDOTHELIAL\_CELL\_DIFFERENTIATION |
| Enrichment Score (ES) | -0.48722595 |
| Normalized Enrichment Score (NES) | -1.7440987 |
| Nominal p-value | 0.0035778175 |
| FDR q-value | 0.1187861 |
| FWER p-Value | 0.999 |
Table: GSEA Results Summary

  

Fig 1: Enrichment plot: GOBP\_REGULATION\_OF\_ENDOTHELIAL\_CELL\_DIFFERENTIATION      
 Profile of the Running ES Score & Positions of GeneSet Members on the Rank Ordered List

  

| SYMBOL | TITLE | RANK IN GENE LIST | RANK METRIC SCORE | RUNNING ES | CORE ENRICHMENT || 1 | TMEM100 | transmembrane protein 100 [Source:HGNC Symbol;Acc:HGNC:25607] | 389 | 0.710 | 0.0262 | No |
| 2 | TNF | tumor necrosis factor [Source:HGNC Symbol;Acc:HGNC:11892] | 2098 | 0.350 | -0.0788 | No |
| 3 | ID1 | "inhibitor of DNA binding 1, HLH protein [Source:HGNC Symbol;Acc:HGNC:5360]" | 3349 | 0.216 | -0.1588 | No |
| 4 | PLCB1 | phospholipase C beta 1 [Source:HGNC Symbol;Acc:HGNC:15917] | 3724 | 0.183 | -0.1733 | No |
| 5 | ADD1 | adducin 1 [Source:HGNC Symbol;Acc:HGNC:243] | 3872 | 0.167 | -0.1714 | No |
| 6 | IKBKB | inhibitor of nuclear factor kappa B kinase subunit beta [Source:HGNC Symbol;Acc:HGNC:5960] | 5914 | 0.001 | -0.3300 | No |
| 7 | FOXJ2 | forkhead box J2 [Source:HGNC Symbol;Acc:HGNC:24818] | 6411 | -0.036 | -0.3657 | No |
| 8 | CEACAM1 | CEA cell adhesion molecule 1 [Source:HGNC Symbol;Acc:HGNC:1814] | 6960 | -0.077 | -0.4022 | No |
| 9 | ETV2 | ETS variant transcription factor 2 [Source:HGNC Symbol;Acc:HGNC:3491] | 7365 | -0.108 | -0.4250 | No |
| 10 | VCL | vinculin [Source:HGNC Symbol;Acc:HGNC:12665] | 7687 | -0.133 | -0.4394 | No |
| 11 | PROC | "protein C, inactivator of coagulation factors Va and VIIIa [Source:HGNC Symbol;Acc:HGNC:9451]" | 7786 | -0.140 | -0.4359 | No |
| 12 | BMP4 | bone morphogenetic protein 4 [Source:HGNC Symbol;Acc:HGNC:1071] | 7816 | -0.142 | -0.4268 | No |
| 13 | ZEB1 | zinc finger E-box binding homeobox 1 [Source:HGNC Symbol;Acc:HGNC:11642] | 8147 | -0.166 | -0.4392 | No |
| 14 | S1PR2 | sphingosine-1-phosphate receptor 2 [Source:HGNC Symbol;Acc:HGNC:3169] | 8154 | -0.167 | -0.4264 | No |
| 15 | TNFRSF1A | TNF receptor superfamily member 1A [Source:HGNC Symbol;Acc:HGNC:11916] | 8196 | -0.171 | -0.4161 | No |
| 16 | XDH | xanthine dehydrogenase [Source:HGNC Symbol;Acc:HGNC:12805] | 8366 | -0.183 | -0.4147 | No |
| 17 | VEZF1 | vascular endothelial zinc finger 1 [Source:HGNC Symbol;Acc:HGNC:12949] | 8621 | -0.202 | -0.4183 | No |
| 18 | GDF2 | growth differentiation factor 2 [Source:HGNC Symbol;Acc:HGNC:4217] | 9223 | -0.250 | -0.4452 | No |
| 19 | F11R | F11 receptor [Source:HGNC Symbol;Acc:HGNC:14685] | 9765 | -0.297 | -0.4636 | Yes |
| 20 | CLDN5 | claudin 5 [Source:HGNC Symbol;Acc:HGNC:2047] | 9816 | -0.302 | -0.4435 | Yes |
| 21 | CDH5 | cadherin 5 [Source:HGNC Symbol;Acc:HGNC:1764] | 9946 | -0.316 | -0.4284 | Yes |
| 22 | BMP6 | bone morphogenetic protein 6 [Source:HGNC Symbol;Acc:HGNC:1073] | 10021 | -0.323 | -0.4085 | Yes |
| 23 | ROCK2 | Rho associated coiled-coil containing protein kinase 2 [Source:HGNC Symbol;Acc:HGNC:10252] | 10648 | -0.388 | -0.4264 | Yes |
| 24 | JAG1 | jagged canonical Notch ligand 1 [Source:HGNC Symbol;Acc:HGNC:6188] | 10977 | -0.427 | -0.4180 | Yes |
| 25 | PPP1R12A | protein phosphatase 1 regulatory subunit 12A [Source:HGNC Symbol;Acc:HGNC:7618] | 11259 | -0.465 | -0.4028 | Yes |
| 26 | BTG1 | BTG anti-proliferation factor 1 [Source:HGNC Symbol;Acc:HGNC:1130] | 11267 | -0.466 | -0.3664 | Yes |
| 27 | NOTCH4 | notch receptor 4 [Source:HGNC Symbol;Acc:HGNC:7884] | 11441 | -0.495 | -0.3405 | Yes |
| 28 | ROCK1 | Rho associated coiled-coil containing protein kinase 1 [Source:HGNC Symbol;Acc:HGNC:10251] | 12046 | -0.605 | -0.3394 | Yes |
| 29 | IL1B | interleukin 1 beta [Source:HGNC Symbol;Acc:HGNC:5992] | 12105 | -0.622 | -0.2946 | Yes |
| 30 | ACVRL1 | activin A receptor like type 1 [Source:HGNC Symbol;Acc:HGNC:175] | 12242 | -0.656 | -0.2531 | Yes |
| 31 | NOTCH1 | notch receptor 1 [Source:HGNC Symbol;Acc:HGNC:7881] | 12555 | -0.776 | -0.2158 | Yes |
| 32 | VEGFA | vascular endothelial growth factor A [Source:HGNC Symbol;Acc:HGNC:12680] | 12629 | -0.813 | -0.1569 | Yes |
| 33 | CTNNB1 | catenin beta 1 [Source:HGNC Symbol;Acc:HGNC:2514] | 12866 | -1.119 | -0.0864 | Yes |
| 34 | APOLD1 | apolipoprotein L domain containing 1 [Source:HGNC Symbol;Acc:HGNC:25268] | 12867 | -1.121 | 0.0026 | Yes |
Table: GSEA details [plain text format]

  

Fig 2: GOBP\_REGULATION\_OF\_ENDOTHELIAL\_CELL\_DIFFERENTIATION      
 Blue-Pink O' Gram in the Space of the Analyzed GeneSet

  

Fig 3: GOBP\_REGULATION\_OF\_ENDOTHELIAL\_CELL\_DIFFERENTIATION: Random ES distribution      
 Gene set null distribution of ES for **GOBP\_REGULATION\_OF\_ENDOTHELIAL\_CELL\_DIFFERENTIATION**
